# Predicting the Severity of Adverse Events on Osteoporosis Drugs Using Attribute Weighted Logistic Regression

**DOI:** 10.3390/ijerph20043289

**Published:** 2023-02-13

**Authors:** Neveen Ibrahim, Lee Kien Foo, Sook-Ling Chua

**Affiliations:** Faculty of Computing and Informatics, Multimedia University, Cyberjaya 63100, Malaysia

**Keywords:** logistic regression, attribute weight, chi-square, osteoporosis disease, adverse drug events

## Abstract

Osteoporosis is a serious bone disease that affects many people worldwide. Various drugs have been used to treat osteoporosis. However, these drugs may cause severe adverse events in patients. Adverse drug events are harmful reactions caused by drug usage and remain one of the leading causes of death in many countries. Predicting serious adverse drug reactions in the early stages can help save patients’ lives and reduce healthcare costs. Classification methods are commonly used to predict the severity of adverse events. These methods usually assume independence among attributes, which may not be practical in real-world applications. In this paper, a new attribute weighted logistic regression is proposed to predict the severity of adverse drug events. Our method relaxes the assumption of independence among the attributes. An evaluation was performed on osteoporosis data obtained from the United States Food and Drug Administration databases. The results showed that our method achieved a higher recognition performance and outperformed baseline methods in predicting the severity of adverse drug events.

## 1. Introduction

Osteoporosis is a common and dangerous bone disease that can lead to serious pain, disability, hospitalization, or even death. According to the International Osteoporosis Foundation [1], older people and women over the age of 50 are at the greatest risk of developing osteoporosis due to physiological changes that come with aging. To date, this disease has affected 200 million people worldwide, and it is expected to increase in the next 5 to 10 years. Although there are a range of drugs used to treat osteoporosis, they may cause various adverse events. An adverse drug event is defined as an injury that affects a patient due to medical intervention linked to drug. Some adverse events are life-threatening and require medical intervention.

There are studies that attempt to investigate adverse events caused by osteoporosis drugs [2,3]. Classification methods are commonly applied to predict adverse events, where data instances are mapped into one of the possible classes. The majority of these studies assume that all attributes are equally important and have the same contribution to the classification decision [4,5,6]. Such an assumption, however, may not be practical in real-world applications. Methods based on attribute weights have been proposed to relax the independence assumption. This approach assigns a continuous value to each attribute, in which the more significant attribute has a higher weight. Some attribute weighting methods have been successfully implemented in a naïve Bayes classifier [7].

Logistic regression (LR) is one of the most widely used classifiers in the biomedicine domain. The maximum-likelihood estimation is used to determine the probability of class membership in LR [8]. However, there are limited studies that apply attribute weights in LR. Current studies applied LR directly on unweighted attributes, which may result in biased estimates and fall short in predicting adverse events [9,10]. In this paper, a new attribute weighted logistic regression is proposed to predict the severity of an adverse osteoporosis drug event. Our contribution is twofold. First, we propose a method to incorporate attribute weights into LR. Second, we present a method to calculate the attribute weights. Our method takes into account the relevance of each attribute in predicting the severity, which not only reduces the impact of irrelevant attributes but also improves the classification performance. We evaluated our method on an osteoporosis adverse events dataset obtained from the U.S. Food and Drug Administration. We have also compared our method with baseline methods.

The outline of this paper is organized as follows: In the next section, we discuss the related work on attribute weighting methods. Section 3 presents our proposed method. Section 4 describes the osteoporosis dataset used in this study. Section 5 presents the experimental results and discussion. Section 6 concludes our findings.

## 2. Related Work

Attribute weight is a continuous value that represents the importance of each attribute in classification. In [11,12,13], the information gain (IG) measure was used to calculate the attribute weights. In the study of [4], their IG-based attribute weight has resulted in some negative values. Ideally, when assigning a weight to an attribute, the weight should not be a negative value.

There are works that used Kullback–Leibler divergence (KL) to calculate the attribute weights for a naïve Bayes classifier [4,5,14]. However, the KL-based attribute weighting method has a longer computational time as this method involves complex calculation steps, including the estimation of weight for each category, the average attribute weight, split information, split weight, and normalized weight, as described in [4].

Ouyed et al. [15] proposed an attribute weighting technique based on the Newton–Raphson method for multi-nominal kernel logistic regression. In this study, each attribute’s relevance to classification is estimated using the Newton–Raphson method. Instead of estimating individual attribute weights for multi-nominal kernel logistic regression, ref. [16] extended the method to allow the estimation of group attribute weights by using gradient descent minimization. Such a method, which uses multiple kernel functions, increases the complexity of the optimization when the data size is large.

Although LR is a widely applied classification method, there are limited studies that incorporate the attribute weights in LR. In some of the studies of LR, the attributes were weighted to perform attribute selection by considering the most relevant attributes [17,18,19,20,21,22]. Krishnapuram et al. [17] introduced a sparse multi-nominal logistic regression to perform automatic attribute selection. In this study, irrelevant attributes with weights equal to zero were removed. Ryali et al. [18] developed a new whole-brain classification method based on sparse logistic regression. Their method combined L1 and L2 norm regularizations to reduce the weight of irrelevant attributes for better attribute selection. Liang et al. [19] investigated the L1/2 penalty with sparse logistic regression for gene selection in cancer prediction. In recent studies by Bertsimas et al. [20,21,22], they reformulated the sparse regression problem on a larger dataset. Their proposed binary reformulation provides sparser classifiers with similar accuracy as the Lasso regularization technique [23]. However, it was indicated in the study that their method is not computationally efficient, especially on a smaller dataset.

Machine learning techniques have been implemented for drug discovery. Lin et al. [24] compared four machine learning models (logistic regression (LR), support vector machine (SVM), random forest (RF), and artificial neural network (ANN)) for personalized treatment of osteoporosis. For testing the generalizability of the models, the main analysis (196 patients) and subgroup analysis (154 patients) were conducted. A genetic algorithm was used to select informative attributes of osteoporosis patients treated in a Taiwan hospital. The grid search method was applied to tune the hyperparameters of SVM, RF, and ANN. In terms of accuracy and precision, there were no differences between the four methods. Neveen et al. [2] applied multi-label classification methods to detect adverse events on the Fosamax drug. Their results showed that decision trees (DT) with classifier chains have better recognition and computational performance compared to SVM and naïve Bayes. Jaganathan et al. [25] used the SVM to predict drug toxicity. Pearson correlation was applied to remove redundant and irrelevant attributes. Recursive feature elimination and cross-validation techniques were used to select the most significant attributes. They tuned their SVM using the grid search method. The hyperparameter-tuned SVM achieved better accuracy and f-score. In another study by Cano et al. [26], they performed RF in two ways: one for attribute ranking and selection and the other for detecting the activity of different drugs based on their chemical compounds. The optimal values of RF parameters were selected based on the lowest prediction error. The results of tuned RF on selected attributes outperformed the results of SVM and multi-layer perceptrons.

Table 1 provides a summary of studies using the attribute weighting method and the attribute selection method.

## 3. Proposed Method

Our method is described in two parts: Section 3.1 describes our approach to incorporating attribute weights into LR, while Section 3.2 describes our approach to calculating attribute weights based on the chi-square statistic.

### 3.1. Weighted Logistic Regresion

Logistic regression is a classification method to predict the logit of a class *Y* from one or more independent attributes as follows:

(1) logit(*Y*) = *α* + *β*_1_*x*_1_ + *β*_2_*x*_2_ + … + *β_n_x_n_*

where *α* is the intercept, *x_i_* (*i* = 1, …, *n*) is the attributes, and *β_i_* (*i* = 1, …, *n*) is the log odds ratios. Both *α* and *β_i_* are estimated using the maximum-likelihood method, which converts to a probability of belonging to a class *Y* as follows:

(2) $$\mathrm{Probability}(Y)=\frac{e^{\alpha +\beta_{1x1}+\beta_{2x2}+\ldots +\beta_{nxn}}}{{1+e}^{\alpha +\beta_{1x1}+\beta_{2x2}+\ldots +\beta_{nxn}}}\;$$

Our method incorporates the attribute weight *w_i_* of attribute *x_i_* as term *a*ln(*w_i_*) into the LR model as follows:

logit(*Y*) = *α* + (*β*_1_ + *a*ln(*w*_1_)) × *x*_1_ + (*β*_2_ + *a*ln(*w*_2_)) × *x*_2_ + … + (*β_n_* + *a*ln(*w_n_*)) × *x_n_* (3)

where *a* denotes a positive or negative sign and ln(*w_i_*) is the natural logarithm value of *w_i_*.

The coefficient *β_i_* in logistic regression is the estimated log odds ratio obtained for a unit change in attribute *x_i_*. The *β_i_* value determines the type of relationship between *x_i_* and the logit of *Y*. If *β_i_* is positive, larger *x_i_* values are associated with a larger logit of *Y*. Conversely, if *β_i_* is negative, larger *x_i_* values are associated with a smaller logit of *Y* [27]. Since ln(*w_i_*) is negative when *w_i_* is less than 1, we proposed to incorporate the weights differently for different combinations of *β_i_* and *w_i_*, as shown in Table 2. For the cases where (1) *β_i_* is negative with *w_i_* < 1 and (2) *β_i_* is positive with *w_i_* > 1, the weight is incorporated by adding a positive ln(*w_i_*) to *β_i_*. For the cases where (3) *β_i_* is negative with *w_i_* > 1 and (4) *β_i_* is positive with *w_i_* < 1, the weight is incorporated by adding a negative ln(*w_i_*) to *β_i_*. By adding the attribute weights as proposed, the intrinsic relationship between *x_i_* and the logit of *Y* is maintained.

Table 3 shows an example of the resulting parameter values for different combinations of *β_i_* and *w_i_*. Referring to the example in Table 3, attribute *x*_1_ has a negative *β* value, while attribute *x*_2_ has a positive *β* value. For *x*_1_, if the attribute weight is larger than 1, the weight is incorporated into the model by adding a negative ln(*w*). Conversely, if the weight of *x*_1_ is less than 1, the weight is incorporated by adding a positive ln(*w*). For *x*_2_, we add positive ln(*w*) to *β* if the weight of *x*_2_ is larger than 1, and negative ln(*w*) if the weight is less than 1. By incorporating weights as proposed, the sign of the resulted coefficient, which represents the log odds ratio of the attributes, remains unchanged, and the magnitude of the weight contribution can be incorporated correctly.

### 3.2. Attribute Weight Based on Chi-Square

Chi-square (χ^2^) is a statistic used in various hypothesis tests. One of them is to test if two categorical attributes are dependent. We propose measuring the weight of an attribute by calculating the χ^2^ value between this attribute and the target attribute. Given the target attribute *T* with classes *t_k_* (*k* = 1, …, *z*) and an attribute *x_i_* with values *b_j_* (*j* = 1, …, *s*), the joint distribution of *T* and *x_i_* is shown in Table 4.

*O_kj_* is the observed number of attribute value *b_j_* that belongs to class *t_k_*, *M_Rk_* is the sum of each row, *M_Cj_* is the sum of each column, and *M* is the total sample size.

The χ^2^ statistic of attribute *x_i_* is calculated as follows:

(4) $$\chi_{i}^{2}\;=\sum \frac{{{(O}_{kj}{\;-\;E}_{kj})}^{2}}{E_{kj}}$$

(5) $$\mathrm{with}\;E_{kj\;}=\frac{M_{Rk}{\times \;M}_{Cj}}{M}$$

*E_kj_* is the expected number of attribute value *b_j_* that belongs to class *t_k_*.

The final weight *w_i_* for attribute *x_i_* based on χ^2^ is computed as follows:

(6) $$w_{i}=\frac{\chi_{i}^{2}\;\times \;n}{\sum_{i=1\;}^{n}\chi_{i}^{2}}\;$$

where *n* is the total number of attributes.

Algorithm 1 shows our proposed attribute weighted logistic regression, where the weights are calculated using χ^2^ measure. First, the attribute weight in the training dataset is calculated using χ^2^ measure. Then, these attribute weights are incorporated to train the LR model (Equation (3)).
**Algorithm 1** Attribute weighted logistic regressionInput: training data 1: For each attribute *x_i_* in the training data    - Compute $\chi_{i}^{2}$ following Equation (4)    - Compute *w_i_* following Equation (6) 2: Incorporate attribute weights to train the weighted LR model (following Equation (3))   If *w_i_* = 0 then set *a*ln(*w_i_*) = 1 × 10^−10^     Else if (*β_i_* > 0 and *w_i_* > 1) or (*β_i_* < 0 and *w_i_* < 1) then *a* = positive     Else if (*β_i_* > 0 and *w_i_* < 1) or (*β_i_* < 0 and *w_i_* > 1) then *a* = negative

## 4. Dataset and Evaluation Methods

This section describes the dataset, data preparation, and evaluation methods used in this study.

### 4.1. Description of the Data

The dataset used in this study was obtained from the online U.S. Food and Drug Administration database from 2004 to 2018 [28]. The data files included in our study are patients’ demographics, drugs, indication (disease), outcome, and therapy. These files are linked via patient ID.

There are 228 drugs reported for adverse events in this dataset. The top ten drugs that were reported as the primary suspects with the most reported adverse events were included in this study. The resulting dataset has 20,576 records with 36 attributes. In this study, we included attributes that are directly related to patient characteristics (age and gender), the drug that caused the adverse event, drug regimens (dose amount, dose unit (microgram or milligram), and dose frequency), the therapy start date, the date of the adverse event, and the stage of osteoporosis disease. There are three stages of osteoporosis disease, which is measured by a Dual-energy X-ray Absorptiometry machine. Osteopenia (pre-osteoporosis) is the first stage and happens when bone density is between −1.5 and −2.5. The second stage is osteoporosis, in which bone density is −2.5. The third stage is the patients who used the related drugs for protection (osteoporosis prophylaxis).

According to the World Health Organization, a “severe adverse event” concerns the critical cases of patients who need immediate medical consultation, for instance, death, disability, hospitalization, or life-threatening conditions. Otherwise, the event is considered non-severe. Following this definition, we have divided the target attribute into two categories—severe and non-severe. The final dataset used in our study has 11,956 severe events and 8620 non-severe events.

### 4.2. Data Preparation

**Figure 1:**
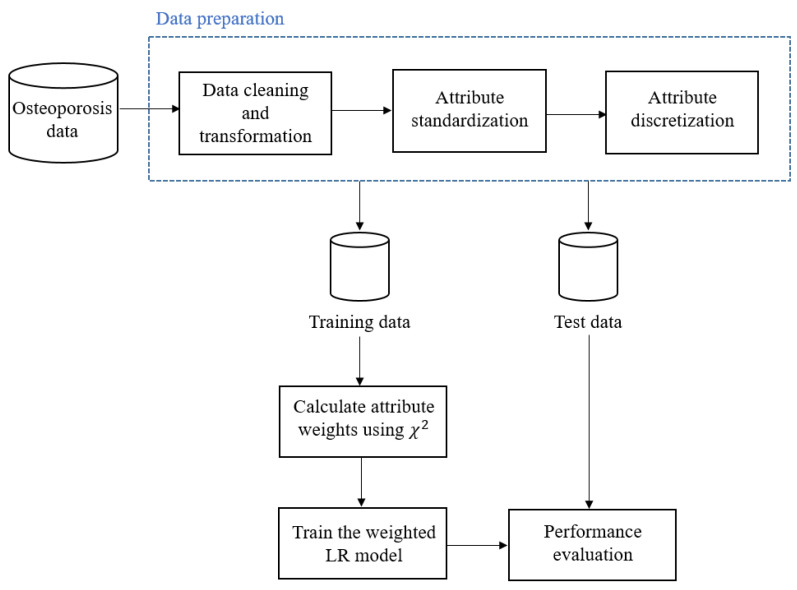
An overview of our method.

For each record, we calculated the number of days between the start of therapy and the occurrence of the adverse event and labelled this as “duration”. Since the dose amounts were reported in milligrams or micrograms, we have converted those dose amounts from milligrams to micrograms. We have standardized the distribution of the three continuous attributes (i.e., age, duration, and dose amount) to have a mean of 0 and a unit standard deviation to avoid bias towards attributes with a large range. For attribute weights calculation, attributes with continuous values have to be discretized [4,5,6,13,29,30,31,32]. These three continuous attributes were discretized by applying the Minimum Description Length method [33]. The process of discretization starts by sorting continuous values in ascending order and then evaluating each candidate cut point, which is the midpoint between each successive pair of data. For cut-point evaluation, the data are divided into two partitions, and the resulting class information entropy is estimated. Finally, the cut-point that has the minimum entropy among all potential cut-points will be chosen to discretize the continuous attributes [33]. As a result of discretization, both age and duration attributes have been converted to categories. The dose amount is excluded from this study as there are no cut-points and all the records belong to the same interval after discretization. Table 5 shows the list of attributes used in this study. An overview of our method is shown in Figure 1.

### 4.3. Evaluation Methods

The classification performance was measured in terms of accuracy, precision, recall, and F-score. The severe class is considered the positive class. Following the definition in [34], accuracy is the ratio of correct predictions, precision is the ratio of positive class predictions that actually belong to the positive class, recall is the ratio of positive class predictions out of all positive records, and F-score is the mean between the precision and the recall.

Accuracy = (True Positives + True Negatives)/All

Precision = True Positives/(True Positives + False Positives)

Recall = True Positives/(True Positives + False Negatives)

F-score = (2 × Precision × Recall)/(Precision + Recall)

## 5. Experiments and Results

The performance of our method was evaluated on the osteoporosis dataset (described in Section 4.1). First, the weights of the attributes were calculated from the training data. Table 6 shows the attribute weights (following Equation (6)) across the 10-fold. These weights are then incorporated into LR.

We have conducted four experiments. The first experiment compared the classification performance of our method against the standard LR, i.e., without applying any attribute weighting method. The second experiment compared our proposed χ^2^ attribute weights with two baseline attribute weighing measures: the KL-based attribute weights [4] and the IG-based attribute weights [12]. The third experiment compared our method with three baseline classification algorithms, i.e., random forest, support vector machine, and decision tree. The fourth experiment compared the computational times of our method and all other baseline methods.

The training set is prepared using the balanced sampling technique, in which we randomly selected 7000 severe and 7000 non-severe records. The remainder (i.e., 6576) is used for testing. The training-test ratio is approximately 70:30. The severe adverse event is defined as a true positive, and we have carried out 10-fold cross-validation for each experiment. The results are presented using comparative boxplots.

### 5.1. Proposed Method against the Standard Logistic Regression

**Figure 2:**
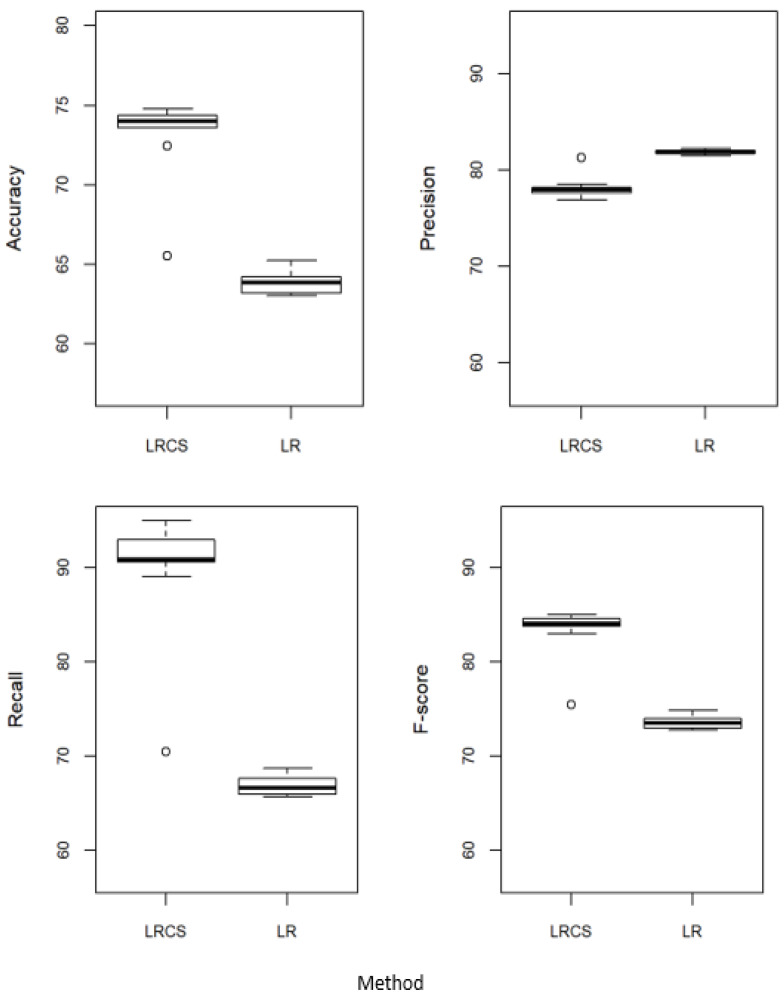
Performance of our method against the standard logistic regression. LRCS: χ^2^ weighted logistic regression. LR: Standard logistic regression without attribute weights.

Figure 2 compares the performance of our method, the χ^2^ weighted logistic regression (LRCS), with the standard logistic regression (LR). LRCS outperformed LR in accuracy, recall, and F-score. The performance of LRCS is about 10% better than that of LR in accuracy and F-score and 20% better in recall. In terms of precision, LR performed slightly better than LRCS.

### 5.2. Proposed Method against the Baseline Attribute Weighing Methods

**Figure 3:**
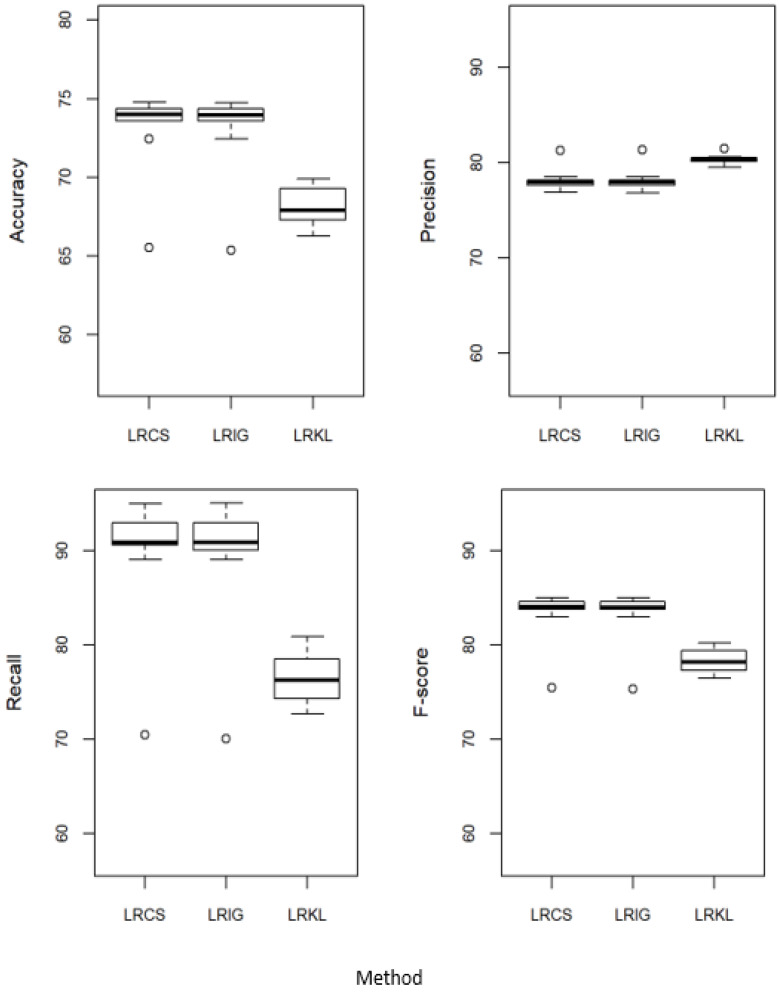
Performance of χ^2^ weighted logistic regression (LRCS) against the baseline attribute weighing measures—IG weighted logistic regression (LRIG) and KL weighted logistic regression (LRKL).

Figure 3 compares the performance of LRCS with two baseline attribute weighing measures, i.e., the weights calculated using IG (LRIG) and the weights calculated using KL (LRKL). These weights are incorporated into LR. Referring to Figure 3, LRCS performed equally to LRIG in all the measures. When comparing to LRKL, our method performed better in accuracy, recall and F-score, but not as good in precision.

### 5.3. Proposed Method against the Baseline Classification Methods

**Figure 4:**
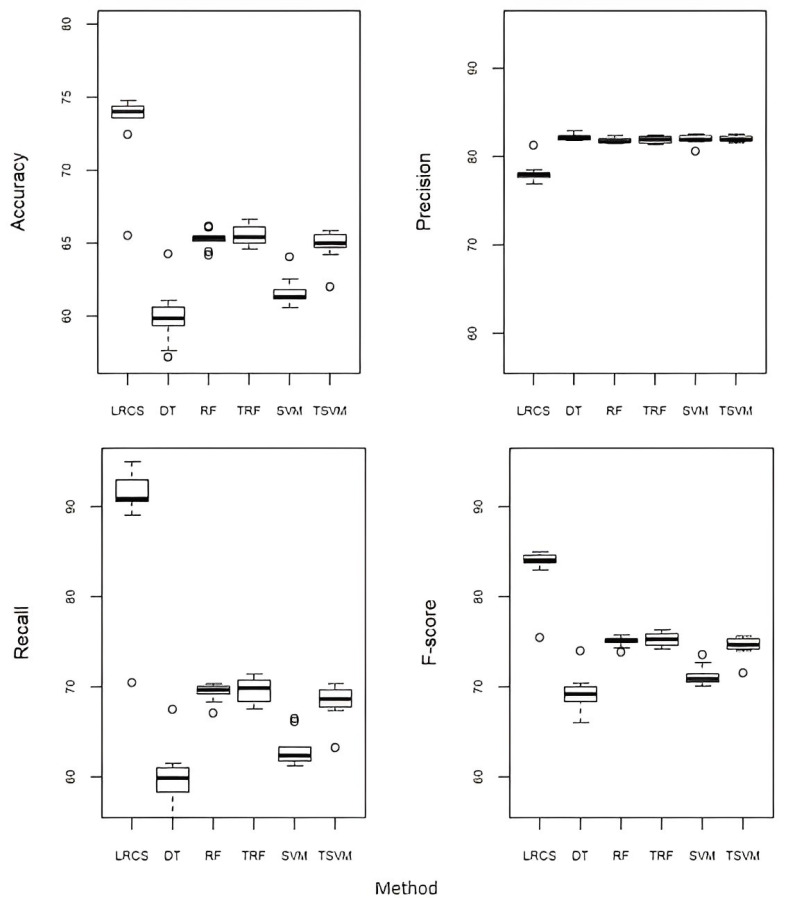
Performance of our method (LRCS) against the baseline classification methods (decision tree (DT), random forest (RF), tuned random forest (TRF), support vector machine (SVM), and tuned support vector machine (TSVM)).

Figure 4 compares the performance of LRCS with three baseline classification methods, i.e., decision tree (DT), random forest (RF), and support vector machine (SVM). Following the approach taken in [26,35], we tuned both the RF and SVM using the grid search method on our training data. For the tuned RF (TRF), the optimal number of trees was 1000, and the optimal number of splits was 2. For the tuned SVM (TSVM), the optimal values for cost and gamma were 0.5. LRCS performed better compared to all the five baseline methods in terms of accuracy (8–15% higher), recall (20–30% higher), and F-score (8–15% higher), but has a slightly lower precision (about 3% lower).

### 5.4. Computational Performance

**Figure 5:**
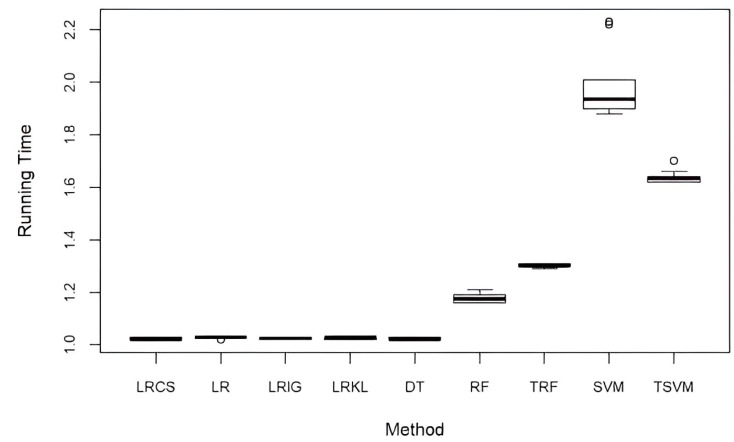
Computational performance of our method and baseline methods (in mins).

Figure 5 shows the running time of LRCS against all the baseline methods. Both the runtimes of TSVM and TRF were calculated after the tuning process was complete. The tuning of SVM took about 1.5 days, while the tuning of RF took about 30 min. All the experiments were performed using RStudio (ver. 1.1.453) on a desktop computer with an Intel Core i5-6500 3.2 GHz and 8 GB RAM. The standard LR and all weighted LR (LRCS, LRIG, and LRKL) have comparable computational times. These results showed that our method to incorporate attribute weights into LR does not increase the computational time. In comparison to the five baseline classification methods (DT, RF, TRF, SVM, and TSVM), LRCS has a comparable computational time with DT, while RF, TRF, SVM, and TSVM have a longer running time. The running time of SVM is almost twofold longer than LRCS.

## 6. Conclusions

In this study, we have proposed: (1) an attribute weight measure based on the chi-square statistic; and (2) a method to incorporate attribute weights into logistic regression to predict the severity of adverse drug events. Experimental results showed that by incorporating attribute weights, the classification performance of logistic regression has improved. Our χ^2^ attribute weights method performed better than the standard logistic regression and KL-based attribute weights, and equally well with the IG-based attribute weights. Our attribute weighted logistic regression performed better than the three baseline methods, i.e., decision tree, random forest, and support vector machine. Our method also outperformed the hyperparameter-tuned random forest and support vector machine. In terms of running time, our method does not affect the computational performance of logistic regression, and the running time is lower compared to random forest, support vector machine, and hyperparameter-tuned models. To the best of our knowledge, this is the first study to propose attribute weighted logistic regression to incorporate the significance of attributes for binary classification. Adverse drug events are sometimes unavoidable, but serious events should be reduced to safeguard patients’ health. The experimental results showed that our method performed well in predicting serious adverse drug events in osteoporosis disease, as the recall of our method is the highest, with an increase of at least 15% compared to all other baseline methods. As for future work, we plan to extend our method to other medical datasets.

## Figures and Tables

**Table 1:** Summary of studies using (**a**) attribute weighting method and (**b**) attribute selection method.

(a)				
Study	Attribute Weighting Method	Classification Method	Dataset	Best Performing Model
Zhang et al. [11]	IG and χ^2^ statistic for words weighting	Multinomial NB and weighted NB	Benchmark textual data obtained from WEKA and Amazon website	IG weighted NB
Duan et al. [12]	IG	NB and weighted NB	Benchmark data from UCI database	IG weighted NB
Zhang and Sheng [13]	IG, hill climbing and Markov Chain Monte Carlo	NB, weighted NB and DT	Benchmark data from UCI database	IG with hill climbing weighted NB
Lee et al. [4]	KL	NB, KL weighted NB, Tree Augmented NB, NBTree and DT	Benchmark data from UCI database	KL weighted NB
Lee [5]	KL and DT	NB, KL feature weighted NB, KL value weighted NB, DT weighted NB, logistic, DT, Tree Augmented NB and RF	Benchmark data from UCI database	KL weighted NB
Foo et al. [7]	IG and KL	NB and weighted NB	Benchmark data from UCI and FDA databases	NA
Korkmaz and Korkmaz [14]	KL	KL weighted NB, Bayesian neural network, SVM and neural network	Breast cancer mammography	KL weighted NB
Ouyed and Allili [15]	Newton Raphson method	NB, Sparse multinomial LR, feature relevance multinomial kernel LR, kernel SVM and Lasso	Benchmark data from UCI database and simulated data	Multinominal kernel LR
Ouyed and Allili [16]	Gradient descent minimisation	Multinominal kernel LR, SVM and deep learning network	UT-interaction dataset	Multinominal kernel LR
		**(b)**		
**Study**	**Attribute Selection Method**	**Classification Method**	**Dataset**	**Best Performing Model**
Krishnapuram et al. [17]	L1 regularization	SVM, relevance vector machine, sparse multinomial LR and ridge multinomial LR	Benchmark data from UCI database and online sources	Sparse multinomial LR
Ryali et al. [18]	Combination of L1 and L2 regularization	SVM based recursive feature elimination, LR based L1 and L2	Whole brain dataset	L1 and L2 based LR
Liang et al. [19]	L_1/2_ regularization	*k*-nearest neighbor	Cancer datasets	NA
Lin et al. [24]	Genetic algorithm	LR, SVM, RF, ANN	Osteoporosis patients	LR and ANN
Jaganathan et al. [25]	Recursive feature elimination and cross-validation	SVM, LR, RF, DT, NB, MLP, XG boosting and *k*-nearest neighbor	Drug toxicity	Hyperparameter-tuned SVM
Cano et al. [26]	Random forest	RF, SVM and MLP	Drug activity	Hyperparameter-tuned RF

**Table 2:** Adding attributes’ weight based on *β* value.

*β* *_i_*	Adding Attribute Weight		The Resulted Value
	*w_i_* < 1	*w_i_* > 1	
Negative	+ln(*w_i_*)	−ln(*w_i_*)	Negative
Positive	−ln(*w_i_*)	+ln(*w_i_*)	Positive

**Table 3:** Example of adding attribute weight to different *β* values.

Attribute	*β_i_*	Weight (*w_i_*)	±ln(*w_i_*)	*β_i_* ± ln(*w_i_*)
*x*_1_	−0.751	1.84	−ln(1.84)	−1.36
		0.86	+ln(0.86)	−0.901
*x*_2_	0.262	1.84	+ln(1.84)	0.871
		0.86	−ln(0.86)	0.412

**Table 4:** Joint distribution of target attribute *T* and attribute *x_i_*.

		*x_i_*				
		*b*_1_	*b*_2_	….	*b_s_*	
*T*	*t*_1_	*O*_11_	*O*_12_	….	*O*_1*s*_	*M_R_*_1_
	*t*_2_	*O*_21_	*O*_22_	….	*O*_2*s*_	*M_R_*_2_
	.	.				.
	.	.				.
	.	.				.
	.	.				.
	*t_z_*	*O_z_*_1_	*O_z_*_2_	….	*O_zs_*	*M_Rz_*
		*M_C_*_1_	*M_C_*_2_	….	*M_Cs_*	*M*

**Table 5:** List of attributes in the osteoporosis dataset.

No	Attribute	Description	Attributes Values	Count	Severe Count	Non-Severe Count
1	Disease	A medical terminology based on medical dictionary for regulatory activities	Osteoporosis Osteopenia Osteoporosis prophylaxis	19,622 789 165	11,536 342 78	8086 447 87
2	Gender	Patient’s sex	Female	18,522	10,633	7889
			Male	2054	1323	731
3	Drug name	The name of reported medicine	Forteo	11,980	7929	4051
			Aclasta	1633	863	770
			Zolendronic acid	1270	522	748
			Prolia	1221	508	713
			Reclast	1179	580	599
			Fosamax	1141	564	577
			Actonel	824	441	383
			Evista	544	267	277
			Boniva	519	147	372
			Alendronate sodium	265	135	130
4	Dose frequency	The reported dosage frequency	Once	349	185	164
			Every day	12,788	8364	4424
			Every week	1801	886	915
			Every month	611	192	419
			Every 3 months	74	28	46
			Every 6 months	1043	443	600
			Every year	3910	1858	2052
5	Age	Patient’s age at event date	From 0 to 105 year	20,576		
6	Duration	The period of using the drug until the event occurring	From 0 to 8677 day	20,576		
7	Target attribute	The patient’s outcome of using the drug	Severe	11,956		
			Non-severe	8620		

**Table 6:** The calculated attribute weights across the 10-fold.

Attribute	Attribute Weights Using χ^2^									
	Fold 1	Fold 2	Fold 3	Fold 4	Fold 5	Fold 6	Fold 7	Fold 8	Fold 9	Fold 10
Disease	0.19	0.14	0.15	0.16	0.18	0.17	0.17	0.18	0.17	0.18
Gender	0.07	0.06	0.07	0.09	0.09	0.08	0.05	0.08	0.07	0.07
Drug name	2.07	2.06	2.02	2.08	2.08	1.98	2.07	1.92	1.98	2.07
Dose frequency	1.87	1.83	1.78	1.84	1.89	1.82	1.82	1.71	1.84	1.81
Age	0.75	0.78	0.87	0.72	0.68	0.78	0.72	0.87	0.87	0.76
Duration	1.05	1.13	1.11	1.11	1.08	1.17	1.17	1.24	1.07	1.11

## References

[1] International Osteoporosis Foundation Website. www.iofbonehealth.org.

[2] Ibrahim N., Belal N., Badawy O. (2014). Data mining model to predict Fosamax adverse events. Int. J. Comput. Inf. Technol..

[3] Yildirim P., Ekmekci I.O., Holzinger A. (2013). On knowledge discovery in open medical data on the example of the FDA drug adverse event reporting system for alendronate (Fosamax). International Workshop on Human-Computer Interaction and Knowledge Discovery in Complex, Unstructured, Big Data.

[4] Lee C.H., Gutierrez F., Dou D. Calculating feature weights in naive Bayes with Kullback-Leibler measure. Proceedings of the 11th International Conference on Data Mining.

[5] Lee C.H. (2018). An information-theoretic filter approach for value weighted classification learning in naive Bayes. Data Knowl. Eng..

[6] Lee C.H. (2015). A gradient approach for value weighted classification learning in naive Bayes. Knowl. Based Syst..

[7] Foo L.K., Chua S.L., Ibrahim N. (2022). Attribute weighted naïve Bayes classifier. Comput. Mater. Contin..

[8] Dreiseitl S., Ohno-Machado L. (2002). Logistic regression and artificial neural network classification models: A methodology review. J. Biomed. Inform..

[9] Duan J.Z. (2009). Two Commonly Used Methods for Exposure—Adverse Events Analysis: Comparisons and Evaluations. J. Clin. Pharmacol..

[10] Nam K., Henderson N.C., Rohan P., Woo E.J., Russek-Cohen E. (2017). Logistic regression likelihood ratio test analysis for detecting signals of adverse events in post-market safety surveillance. J. Biopharm. Stat..

[11] Zhang L., Jiang L., Li C., Kong G. (2016). Two feature weighting approaches for naive Bayes text classifiers. Knowl. Based Syst..

[12] Duan W., Lu X.Y. Weighted naive Bayesian classifier model based on information gain. Proceedings of the 2010 International Conference on Intelligent System Design and Engineering Application.

[13] Zhang H., Sheng S. Learning weighted naive Bayes with accurate ranking. Proceedings of the Fourth IEEE International Conference on Data Mining (ICDM’04).

[14] Korkmaz S.A., Korkmaz M.F. (2015). A new method based cancer detection in mammogram textures by finding feature weights and using Kullback–Leibler measure with kernel estimation. Optik.

[15] Ouyed O., Allili M.S. (2018). Feature weighting for multinomial kernel logistic regression and application to action recognition. Neurocomputing.

[16] Ouyed O., Allili M.S. (2020). Group-of-features relevance in multinomial kernel logistic regression and application to human interaction recognition. Expert Syst. Appl..

[17] Krishnapuram B., Carin L., Figueiredo M.A., Hartemink A.J. (2005). Sparse multinomial logistic regression: Fast algorithms and generalization bounds. IEEE Trans. Pattern Anal. Mach. Intell..

[18] Ryali S., Supekar K., Abrams D.A., Menon V. (2010). Sparse logistic regression for whole-brain classification of fMRI data. NeuroImage.

[19] Liang Y., Liu C., Luan X.Z., Leung K.S., Chan T.M., Xu Z.B., Zhang H. (2013). Sparse logistic regression with a L 1/2 penalty for gene selection in cancer classification. BMC Bioinform..

[20] Bertsimas D., Pauphilet J., Parys B.V. (2017). Sparse classification: A scalable discrete optimization perspective. arXiv.

[21] Bertsimas D., Parys B.V. (2020). Sparse high-dimensional regression: Exact scalable algorithms and phase transitions. Ann. Stat..

[22] Bertsimas D., Pauphilet J., Parys B.V. (2020). Sparse regression: Scalable algorithms and empirical performance. Stat. Sci..

[23] Bach F.R. (2008). Consistency of the group lasso and multiple kernel learning. J. Mach. Learn. Res..

[24] Lin Y.T., Chu C.Y., Hung K.S., Lu C.H., Bednarczyk E.M., Chen H.Y. (2022). Can machine learning predict pharmacotherapy outcomes? An application study in osteoporosis. Comput. Methods Programs Biomed..

[25] Jaganathan K., Tayara H., Chong K.T. (2021). Prediction of drug-induced liver toxicity using SVM and optimal descriptor sets. Int. J. Mol. Sci..

[26] Cano G., Garcia-Rodriguez J., Garcia-Garcia A., Perez-Sanchez H., Benediktsson J.A., Thapa A., Barr A. (2017). Automatic selection of molecular descriptors using random forest: Application to drug discovery. Expert Syst. Appl..

[27] Peng C.Y.J., Lee K.L., Ingersoll G.M. (2002). An introduction to logistic regression analysis and reporting. J. Educ. Res..

[28] US FDA Database Website. https://fis.fda.gov/extensions/FPD-QDE-FAERS/FPD-QDE-FAERS.html.

[29] Taheri S., Yearwood J., Mammadov M., Seifollahi S. (2014). Attribute weighted Naive Bayes classifier using a local optimization. Neural Comput. Appl..

[30] Frank E., Hall M., Pfahringer B. (2022). Locally weighted naive bayes. In Proceedings of the Nineteenth Conference on Uncertainty in Artificial Intelligence. arXiv.

[31] Jiang L., Zhang L., Li C., Wu J. (2018). A correlation-based feature weighting filter for naive Bayes. IEEE Trans. Knowl. Data Eng..

[32] Jiang L., Li C., Wang S., Zhang L. (2016). Deep feature weighting for naive Bayes and its application to text classification. Eng. Appl. Artif. Intell..

[33] Fayyad U., Irani K. Multi-interval discretization of continuous-valued attributes for classification learning. Proceedings of the 13th International Joint Conference on Artificial Intelligence.

[34] Tharwat A. (2021). Classification assessment methods. Appl. Comput. Inform..

[35] Hsu C.W., Chang C.C., Lin C.J. A Practical Guide to Support Vector Classification. https://www.csie.ntu.edu.tw/~cjlin/papers/guide/guide.pdf.

